# Amphiphilic Polymer Nanoreactors for Multiple Step, One-Pot Reactions and Spontaneous Product Separation

**DOI:** 10.3390/polym13121992

**Published:** 2021-06-18

**Authors:** Andrew Harrison, Christina Tang

**Affiliations:** Department of Chemical and Life Sciences Engineering, Virginia Commonwealth University, Richmond, VA 23284-3028, USA; harrisona3@vcu.edu

**Keywords:** micelle, nanoreactor, nanoprecipitation, self-assembly, multicomponent reaction

## Abstract

Performing multiple reaction steps in “one pot” to avoid the need to isolate intermediates is a promising approach for reducing solvent waste associated with liquid phase chemical processing. In this work, we incorporated gold nanoparticle catalysts into polymer nanoreactors via amphiphilic block copolymer directed self-assembly. With the polymer nanoreactors dispersed in water as the bulk solvent, we demonstrated the ability to facilitate two reaction steps in one pot with spontaneous precipitation of the product from the reaction mixture. Specifically, we achieved imide synthesis from 4-nitrophenol and benzaldehyde as a model reaction. The reaction occured in water at ambient conditions; the desired 4-benzylideneaminophenol product spontaneously precipitated from the reaction mixture while the nanoreactors remained stable in dispersion. A 65% isolated yield was achieved. In contrast, PEGylated gold nanoparticles and citrate stabilized gold nanoparticles precipitated with the reaction product, which would complicate both the isolation of the product as well as reuse of the catalyst. Thus, amphiphilic nanoreactors dispersed in water are a promising approach for reducing solvent waste associated with liquid phase chemical processing by using water as the bulk solvent, eliminating the need to isolate intermediates, achieving spontaneous product separation to facilitate the recycling of the reaction mixture, and simplifying the isolation of the desired product.

## 1. Introduction

The production of chemicals generally involves multiple catalytic reactions with a corresponding product separation/purification and catalyst separation between each reaction. The ability to perform multiple reactions in “one pot” decreases the number of workups and purifications, as well as the volume of solvent required. These cascade or domino reactions simplify the process of organic synthesis, lower cost, and reduce waste and product purification steps [1,2].

Such cascade reactions can be performed with multifunctional heterogeneous catalysts in order to ease the separation and reuse of the catalyst [1]. Polymer-based systems have been a versatile approach to achieving such multifunctional catalysts [3,4,5,6,7]. One approach has been to precisely control position of the active sites of multiple catalysts when incorporated into a polymer support to ensure that incompatible catalysts are isolated. For example, monomers with acidic and basic catalytic sites were copolymerized to achieve acidic and basic porous polymers. The multifunctional acid-base catalyst was used for the cascade reaction involving two steps of an acid-catalyzed deacetalization followed by a base-catalyzed Knoevanagel condensation reaction [1]. Similar studies have been performed with bottlebrush copolymers with acidic and basic catalytic sites [2]. Acid-base bifunctional crosslinked polymers have also been incorporated into the pores of mesoporous silica and combined with Ni or Ni–Pd alloy nanoparticles. The multifunctional catalyst system was used for the three-step, one-pot reaction of a deacetalization-Knoevenagel condensation reduction reaction [8]. An alternative approach has been incorporating multiple catalysts into a polymer-based nanocarrier. For example, pluoronic nanocarriers were used to coencapsulate two enzyme biocatalysts, formate dehydrogenase and mannitol dehydrogenase, for the conversion of D-fructose to D-mannitol [9].

Micellar systems are particularly promising for performing one-pot reactions because they facilitate the use of water as a bulk solvent, and the micellar structure can provide site isolation [10,11]. For example, shell cross-linked micelles have also been used for the site isolation of incompatible catalysts to facilitate a three-step tandem one-pot reaction performed in water. Upon self-assembly in water, the concentric structure of the micelle is comprised of carboxylic acids in the hydrophilic outermost corona, rhodium-based catalysts in the intermediate crosslinked layer, and basic catalytic sites in the hydrophobic core. The structure of the nanoreactor was designed to produce an ester product through a tandem reaction. [12]. Similar micellar systems encapsulating coporphyrin catalysts in the micelle core with Rh-based catalysts in the shell have also been reported to produce chiral alcohols via a two-step tandem reaction [13]. Similarly, the organocatalytst TEMPO has been incorporated into the shell of a polymer micelle with Rh-based catalysts in the hydrophobic core to facilitate redox catalysis in aqueous media [10].

While one-pot reactions can be performed with two micelle populations [10] or micelles with enzymes [14], it is important to note that engineering two catalysts into a single nanoreactor reduces the reaction time fourfold compared to a one-pot cascade reaction performed using two different nanoreactors [10]. This improvement in performance was attributed to reduced diffusion limitations [10]. However, engineering these micelle systems with multiple catalysts can be challenging and typically requires a new polymer synthesis for each micelle system.

Alternative systems that use a single metal nanoparticle catalyst have also been considered. For example, gold nanoparticles stabilized with polyionic liquids were used for a one-pot cascade reaction of ethyl cyanoacetate and p-nitrobenzaldehyde. Sodium borohydride was then added to reduce the NO_2_ groups. The tandem reaction was performed in one pot [15]. Tandem reactions have also been performed with silver nanoparticles within polymer reactors made from hydrogel bilayers. The encapsulated silver nanoparticles were used for the reduction of the intermediate, p-nitrophenol, to p-aminophenol. The reactor was temperature responsive. No reaction was observed at 30 °C due to the collapsed state of the first layer and low resulting conversion of p-nitrophenyl acetate. No product was formed at 45 °C due to the collapsed state of the second layer resulting in low conversion of the intermediate. At 60 °C, both reactions occurred in tandem.

Further integrating reactions and separation processes could minimize solvent usage. Such an ability would be impactful because traditionally, downstream separation processes account for as much as two thirds of total manufacturing costs [16]. Green process engineering approaches to combine reaction and separation have included reactive membranes as well as combining continuous flow synthesis with nanofiltration. These promising approaches have recently been demonstrated with single-step reactions [16,17,18].

Building on these examples, we aim to establish an approach for performing integrated multiple step one-pot reactions and separation using polymer micelles as an alternative to systems that require the unique synthesis of amphiphilic molecules. In our previous work, we established that flash nanoprecipitation is a useful tool for producing self-assembled polymer nanoreactors with tunable properties, e.g., size [19], catalyst loading [19], and hydrophobic core material [20]. In this work, we aim to demonstrate that the self-assembled amphiphilic nanoreactors containing a single metal nanoparticle catalyst in the hydrophobic core can facilitate multiple step synthesis in one pot and separation of the product. Given that the self-assembly occurs by noncovalent interactions, we are further interested in comparing the performance of the polymer nanoreactor with alternative stabilizers of the catalyst. Specifically, we aim to investigate what advantages the self-assembled polymer structure provides compared to small molecule and hydrophilic ligands. Overall, the novel aspects of this work are demonstrating that the amphiphilic self-assembled nanoreactors containing a single catalyst can facilitate multiple reactions without requiring the unique synthesis of amphiphilic molecules and that the amphiphilic polymer structures perform better than small molecule and hydrophilic ligands.

Specifically, in this proof-of-concept study, we use imine synthesis via a one-pot condensation reaction of benzaldehyde and 4-nitrophenol as a model reaction since imines are an important class of molecules [21,22] that are commonly used for the preparation of heterocycles, anti-inflammatory agents, and anticancer agents, and the reduction of 4-nitrophenol is well studied [23,24]. As a catalyst, we incorporate gold nanoparticles into polystyrene nanoreactors stabilized by polyethylene glycol. The effect of pH on the reaction is discussed. The amphiphilic polymer nanoreactors are compared to gold nanoparticles stabilized by hydrophilic stabilizers such as citrate and PEG.

## 2. Materials and Methods

### 2.1. Materials

Sodium borohydride (commercial grade, >96%), 4-nitrophenol (reagent grade, ≥99%), benzaldehyde (reagent grade, ≥99%), formic acid (reagent grade, ≥95%), sodium hydroxide (ACS reagent grade, ≥97.0%), and potassium chloride (ACS reagent grade, ≥99%) were purchased from Sigma-Aldrich (St. Louis, MO, USA). Solvents: tetrahydrofuran (THF) (HPLC grade, >99.9%), ethanol (ACS reagent grade, 88–91%), and diethyl ether (ACS reagent grade, >99%) were purchased from Fisher Scientific (Fairmont, NJ, USA). Dodecanethiol (DDT)-stabilized 5 nm nanoparticles and polyethylene glycol-stabilized, i.e., PEGylated, 5 nm gold nanoparticles were purchased from Fisher Scientific (Fairmont, NJ, USA). Citrate-stabilized 5 nm gold nanoparticles were purchased from Ted Pella (Redding, CA, USA). The 4-aminophenol (reagent grade, > 98%) was purchased from Tokyo Chemical Industry Co. (Portland, OR, USA). Environmental grade hydrochloric Acid 30–38% and environmental grade nitric acid 70% were purchased from GFS Chemicals (Columbus, OH, USA). The ^1^H-NMR solvent acetone-D6 with 4,4-dimethyl-4-silapentane-1-sulfonic acid (DSS) as an internal standard was purchased from Cambridge Isotope Lab, Inc. (Andover, MA, USA). These chemicals and materials were used as received. Polystyrene (PS, Mw 7500 g/mol), abbreviated PS 7500, and polystyrene-b-polyethylene glycol (PS-b-PEG, PSm-b-PEGn where m = 1600 g/mol and *n* = 5000 g/mol) were obtained from Polymer Source, Inc. (Montreal, Quebec, Canada) (Product No. P13141-SEO). Before using, PS-b-PEG was dissolved in THF (500 mg/mL) and precipitated into a large volume of ether (~1:20 *v*/*v* THF:ether). The purified PS-b-PEG was recovered by centrifuging, decanting, and drying under vacuum at room temperature for 2 days as previously described [19].

### 2.2. Nanoreactor Assembly

Flash nanoprecipitation was used to fabricate amphiphilic polymer nanoreactors similar to previous reports [19]. Briefly, to incorporate hydrophobic gold nanoparticles into amphiphilic polymer nanoreactors, the first step was to transfer dodecanethiol-capped gold nanoparticles (5 nm) from toluene into in a water miscible solvent. Thus, 1 mL of the as-received dodecanethiol-stabilized gold nanoparticles (in toluene) were precipitated into ethanol (45 mL). The particles were filtered, resuspended in THF, and the ethanol was partially evaporated overnight at room temperature to concentrate the particles. To characterize the gold nanoparticles, the final concentration was measured by inductively coupled plasma optical emission spectroscopy (ICP-OES) using an Agilent 5110 (ICP-OES, Santa Clara, CA, USA), and UV absorbance was measured on an Ocean Optics FLAME-S-UV-VIS with a HL-2000-FHSA light source (Largo, FL, USA) before and after the solvent switch to confirm that processing did not significantly affect gold nanoparticle size [19].

To incorporate the gold nanoparticles into polymer nanoreactors, dodecanethiol-stabilized 5 nm gold nanoparticles (1 mg), PS 7500, (11 mg), and PS-b-PEG (12 mg) were added to 1 mL of tetrahydrofuran (THF) and sonicated at 55 °C for 30 min to dissolve the block copolymer polymer and disperse the gold nanoparticles. Nanoreactor assembly was performed using a manually operated confined impinging jet mixer with dilution (CIJ-D) [25,26] and achievable Reynolds’ numbers > 1300. The THF stream containing the block copolymer, coprecipitate, and dispersed gold nanoparticles was rapidly mixed against 1 mL of water and immediately diluted into a stirring vial of water (8 mL). The resulting dispersion (10 mL total) was centrifuged at 14,000 rpm for 20 min, the supernatant separated from the pellet and reconstituted to the original volume with DI water. The supernatant solutions were stored at room temperature for further characterization and analysis.

### 2.3. Nanoreactor Characterization

For characterization, the nanoreactor size (i.e., hydrodynamic diameter) was measured using a Malvern Zetasizer Nano ZS (Westborough, MA, USA) with a backscatter detection angle of 173°. Intensity weighted size distributions with normal resolution are reported (average of four measurements). Unless otherwise noted, the reported size is the peak 1 mean intensity. The polydispersity index (PDI) provides a measure of particle size distribution and is defined from the moment of the cumulant fit of the autocorrelation function calculated by the instrument software (appropriate for samples with PDI < 0.3) [25].

For visualization by TEM, grids were submerged in a dilute dispersion of nanoreactors (10-fold dilution with water) for one hour and dried at ambient conditions overnight. The samples were dried under ambient conditions. Then, the samples were imaged with a JEM-1230 system (JEOL, Peabody, MA, USA) at 120 kV.

To determine the gold loading in the nanoreactors (wt. gold/wt. nanoreactor), the polymer nanoreactor concentration was measured by thermogravimetric analysis (Perkin Elmer Pyris 1 TGA (Waltham, MA, USA) [27]), and the gold concentration was measured by inductively coupled plasma optical emission spectroscopy (ICP-OES). Briefly, to determine the gold concentration, the nanoreactor dispersions were dissolved in THF and digested in aqua regia (1:3 nitric acid:hydrochloric acid by volume) for at least 24 h. The samples were then diluted to 5% *v*/*v* aqua regia. The gold concentration of the digested sample was measured using ICP-OES measurements with an Agilent 5110 (Santa Clara, CA, USA). A matrix modifier, potassium chloride (2 mg/mL) in 5% *v*/*v* aqua regia, was used to increase the ion concentration, which proved beneficial for peak resolution.

### 2.4. One-Pot Reaction

The desired one-pot condensation of benzaldehyde with 4-nitrophenol involves two reaction steps: (1) the reduction of 4-nitrophenol to 4-aminophenol and (2) the condensation of benzaldehyde with 4-aminophenol. In order to perform the reaction in one pot, we first studied the reactions separately.

Following well-established protocols, the reduction of 4-nitrophenol using the gold nanoreactors was studied in the presence of sodium borohydride [23,28]. Typically, nanoreactors (0.0079 mol% AuNP) were added to 4-nitrophenol (20 µL, 0.01 M, abbreviated 4NP) followed by aqueous sodium borohydride (initially 6 M, within 5 min of preparation, abbreviated NaBH_4_) to form a 2-mL reaction solution. The progress of the reaction (i.e., conversion of 4NP) was monitored using UV spectroscopy (Ocean Optics FLAME-S-VIS-NIR-ES, Largo, FL, USA), with a HL-2000-FHSA light source (300–1200 nm) with a CUV-UV cuvette holder placed on a stir plate. The final reaction mixture contained less than 0.01 vol% THF (residual from nanoreactor self-assembly). The induction time and apparent reaction rate (*K_app_*) were determined from monitoring the change in the absorbance at 425 nm as a function of time. The values of *K_app_* and induction time were the averages (±standard deviations) of at least 3 trials of each experiment. The catalyst surface area normalized reaction rate constant (k_1_) was then determined based on previous reports [19].

To study the condensation reaction, benzaldehyde (20 µmol) and 4-aminophenol (18 µmol) were placed in 2 mL of water. The pH was adjusted using the appropriate amounts of formic acid and NaOH (10 M). The reaction solution was allowed to sit overnight, and the solution was analyzed by GC-MS (HP 6890 GC with HP 5973 MSD) with a 5% phenyl methyl siloxane column. If a precipitate was present, the solution was then filtered through a syringe filter to remove the precipitate. The filter cake was resuspended in acetone d-6 (1 mL) and analyzed by NMR to confirm the presence of the desired reaction product.

The one-pot reaction was carried out in a 10-mL conical tube. Unless otherwise noted, polymer nanoreactors (500 µL) were added to 960 µL of water followed by the addition of 4-nitrophenol (500 µL, 4 mg/mL). A fresh solution of sodium borohydride (40 µL, 3.78 mg/mL) was then added to the reaction solution. Once the solution turned colorless, formic acid (20 µL) was added in order to neutralize the remaining sodium borohydride. After bubbling in the reaction solution had subsided, benzaldehyde (20 µmol) was added followed by sodium hydroxide solution (10 M, 30 µL). The reaction solution was allowed to sit overnight, centrifuged at 4000 rpm for 20 min, and decanted to collect the precipitated product. The pellet was dried. The product was extracted in acetone d-6 analyzed by GCMS (HP 6890 GC with HP 5973 MSD) and NMR (Bruker 400 MHz), dried, and weighed. The isolated yield of the product (mass product/mass product possible) was an average of three trials. Any solid that was insoluble in the acetone-d6 extraction was also weighed. The other solid precipitate mass was reported as an average of three trials. Finally, the pellet was digested with 667 µL of aqua regia for 24 h, diluted to 5% *v*/*v* aqua regia, and analyzed by ICP-OES for gold content. A matrix modifier, potassium chloride (2 mg/mL) in 5% *v*/*v* aqua regia, was used to increase the ion concentration to improve peak resolution. We compared the performance of the self-assembled nanoreactors in the one-pot reaction with as-received 5-nm PEGylated gold nanoparticles and 5-nm citrate-stabilized gold nanoparticles using the same mass of gold in the reaction.

## 3. Results and Discussion

Gold nanoparticles were incorporated into amphiphilic polymer nanoreactors using block-copolymer directed self-assembly via flash nanoprecipitation [19,20]. To perform polymer directed self-assembly, hydrophobic gold nanoparticles were dispersed with a dissolved amphiphilic block copolymer (polystyrene-b-polyethylene glycol (PS-b-PEG)) and polystyrene coprecipitant in a water miscible organic solvent (THF) and rapidly mixed with water in a confined impinging jet mixer. Upon mixing with water, the gold nanoparticles aggregated and polystyrene precipitated. Simultaneously, the block copolymer micellized, directing the self-assembly of the nanoreactors. The hydrophobic block of the amphiphilic block copolymer adsorbed to the precipitating nanoreactor components via hydrophobic interactions; the hydrophilic PEG block sterically stabilized the nanoreactor. In previous work, we established the formulation parameters to achieve polystyrene nanoreactors with tunable size and gold loading [19]. Building on this work, our focus was to demonstrate that the polymer nanoreactors dispersed in water could facilitate a multistep reaction in one pot with spontaneous precipitation of the product from the reaction mixture to reduce the solvent waste associated with liquid phase chemical processing.

**Figure 1:**
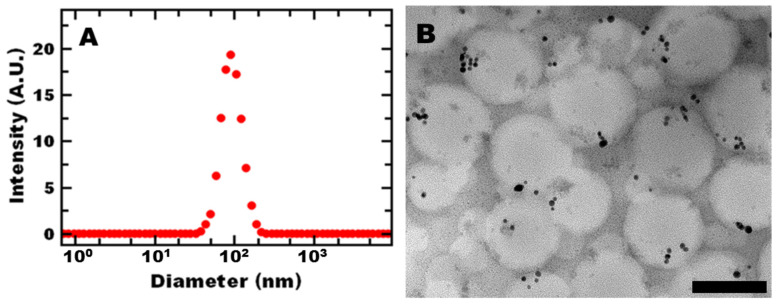
Representative (**A**) DLS (intensity-weighted average of 4 measurements) and (**B**) TEM of PS 7500 nanoreactors with a 100 nm scale bar.

Based on our previous work, we aimed to produce sub-100-nm nanoreactors (through selection of the block copolymer, polystyrene, and gold concentration). At this size, no internal mass transfer limitations were observed [19]. After formulation, the polymer nanoreactors had an average hydrodynamic diameter of 91 ± 5 nm and PDI of 0.163 ± 0.018 by DLS (Figure 1A). Transmission electron microscopy (TEM) (Figure 1B) confirmed that the nanoreactors were spherical and contained gold. The overall nanoreactor size was consistent with DLS measurements. Gold nanoparticles appear to be incorporated in the nanoparticle core. Some gold nanoparticles may also be associated with the PEG shell. Gold unassociated with the nanoreactors would be expected to precipitate out of the dispersion as well as affect the size distribution measured by DLS, which was not observed. These results are consistent with previous reports [19,20].

To further analyze the gold content in the nanoreactors, the gold loading in the nanoreactors was determined by measuring the gold content in the nanoreactor dispersion using inductively coupled plasma optical emission spectrometry (ICP-OES) analysis and thermogravimetric analysis (TGA) to determine the nanoreactor concentration (mg/mL). We note that based on TGA analysis, the nanoreactor concentration was over 80% of the nominal concentration, which was comparable to previous reports of recovery using centrifugal processing [29]. The gold loading was determined to be 1.1 ± 0.1%. (wt. gold/wt. nanoreactors).

Initially, we confirmed that the gold nanoparticles incorporated in the nanoreactors were catalytically active using the reduction of 4-nitrophenol with sodium borohydride. The 4-nitrophenol reduction is a well-known model reaction for studying the activity of gold nanoparticle catalysts [23,24]. We compared the activity of the nanoreactors to citrate-stabilized gold nanoparticles and PEGylated gold nanoparticles under the same gold, 4-nitrophenol, and sodium borohydride conditions. The results are reported in Table 1. At a sodium borohydride concentration of 0.1 M, the polymer nanoreactors demonstrated twofold greater activity than citrate AuNP. The activity of the nanoreactors is slightly lower (0.7-fold) than the PEG AuNP. This demonstrates that incorporating gold nanoparticle catalysts in an amphiphilic polymer nanoreactor is not detrimental to catalytic performance. The enhanced apparent catalytic activity compared to citrate AuNPs could be attributed to reagent solubility differences in the localized reaction environments [6].

When we attempted a one-pot cascade reaction adding all the reactants (4-nitrophenol, sodium borohydride, and benzaldehyde) at the same time, analysis by GC-MS indicated that benzaldehyde reacted with the sodium borohydride, producing benzyl alcohol rather than the desired product. This result is consistent with previous studies [30]. Since the benzaldehyde was converted to benzyl alcohol, the desired cascade reaction of reduction of 4-nitrophenol to 4-aminophenol followed by reaction with benzaldehyde was not achieved.

**Figure 2:**
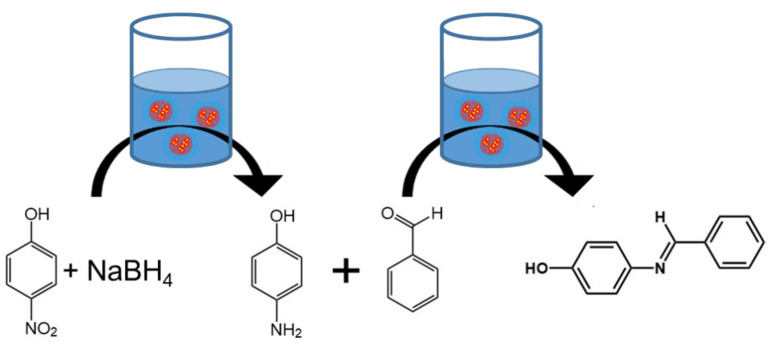
Schematic of the one-pot condensation of benzaldehyde with 4-nitrophenol. First, 4-nitrophenol and sodium borohydride were added to an aqueous dispersion of polymer nanoreactors to produce 4-aminophenol. After the first reaction, the pH was adjusted using formic acid and sodium hydroxide. Finally, addition of benzaldehyde facilitated the condensation of benzaldehyde with 4-aminophenol to produce the desired 4-benzylideneaminophenol product which spontaneously phase separated from the nanoreactor dispersion.

From these results, we determined that the sodium borohydride used for the reduction of 4-nitrophenol caused an unwanted side reaction in the presence of benzaldehyde. Therefore, to perform the two-step reaction in one pot, the reactions needed to be performed sequentially. Specifically, the sodium borohydride needed to be consumed prior to the addition of benzaldehyde. Thus, our approach to this “one pot” reaction was to first add the 4-nitrophenol and sodium borohydride to produce 4-aminophenol, neutralize the nanoreactor dispersion with formic acid, and then add benzaldehyde. An overview of this one-pot approach is provided in Figure 2.

**Figure 3:**
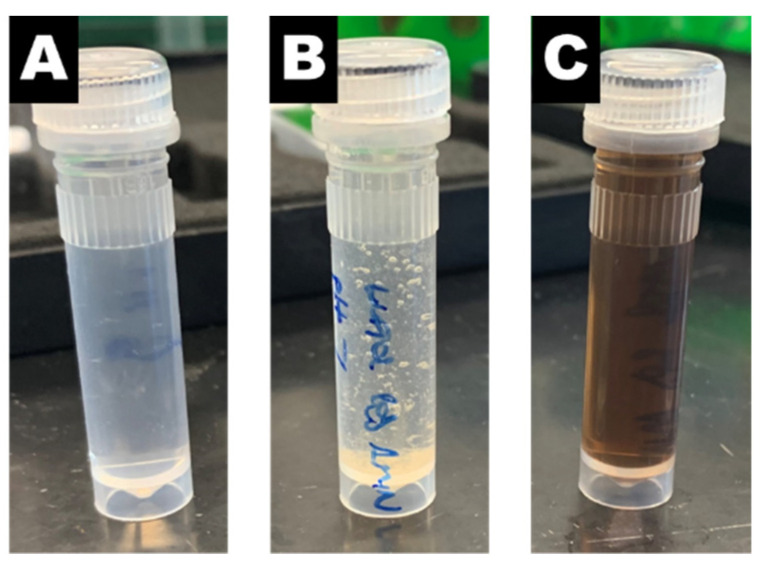
The effect of pH condensation of benzaldehyde with 4-aminophenol. (**A**) Reaction solution pH of 4 with no color change or precipitate observed. (**B**) Reaction solution pH of 7 with a precipitate observed. (**C**) Reaction solution pH of 10 with a brown color changed observed and no precipitate.

To determine the appropriate pH for the second reaction, we examined the effect of pH on the desired reaction between 4-aminophenol and benzaldehyde. Experiments in the condensation of benzaldehyde with 4-aminophenol without nanoreactors (i.e., no gold) showed that the pH of the reaction solution strongly influences the outcome of the reaction. At pH 4, the reaction solution remained colorless with no precipitate formed (Figure 3A). No desired product was detected by GC-MS or NMR. In contrast, increasing the pH to neutral resulted in precipitate formation (Figure 3B). NMR analysis confirmed that the precipitate formed was the desired 4-benzylideneaminophenol product (Figure S1). Further increasing the pH to 10 resulted in a dark brown reaction solution (Figure 3C). The desired product was not detected by either GC-MS or NMR. The color change may be attributed to the oxidation-induced polymerization of 4-aminophenol [31]. From these experiments, the condensation of benzaldehyde and 4-aminopheno does not require gold but does require neutral pH. Therefore, after the reaction between 4-nitrophenol and sodium borohydride, we aimed to ensure that the sodium borohydride was completely consumed and to adjust the pH of the reaction solution to 7 when performing the one-pot reaction with nanoreactors.

To perform the two-step sequential one-pot reaction, first 4-nitrophenol and sodium borohydride were added to the nanoreactor dispersion. Following the 4-nitrophneol reduction to 4-aminophenol, indicated by the color change from yellow to colorless (i.e., 100% conversion of 4-nitrophenol by UV–vis spectroscopy), formic acid was added to neutralize the reaction mixture to ensure decomposition of sodium borohydride via hydrolysis [32]. Then, benzaldehyde was added with small amounts of sodium hydroxide (20 μL of 10 M NaOH) to achieve pH 7 (Figure 2). In this case, there was a color change to light brown associated with the desired reaction as well as a precipitate. The precipitate was characteristic of the condensation of benzaldehyde with 4-aminophenol at neutral pH. Since the desired product is hydrophobic, it spontaneously phase separated from the reaction mixture and could be easily separated from the nanoreactors. We note that at the same conditions we confirmed that no product was formed without nanoreactors because the multistep reaction could not proceed since gold is required for the first reaction.

Following the cascade reaction, the nanoreactor dispersion was centrifuged, the nanoreactor dispersion was decanted, and the resulting pellet was analyzed. The resulting pellet was partially solubilized using deuterated acetone. The components following extraction were analyzed by NMR. NMR analysis confirmed that the pellet contained the desired 4-benzylideneaminophenol product (Figure S1). The mass of product extracted from the pellet was measured (Table 2), and the yield (based on the molar amounts of 4-nitrophenol and benzaldehyde) was 65%. The turnover frequency based on the mass of product, mass of gold, and reaction time was 9 h^−1^. The yield and turnover frequency achieved are comparable to previous reports [33]. Importantly, these results demonstrate that multiple reactions could be achieved in “one pot” using water as the bulk solvent at ambient temperature and pressure. Furthermore, the product spontaneously precipitated from the reaction mixture.

Overall, these results demonstrate that the self-assembled polymer micelle nanoreactors incorporating gold nanoparticle catalysts dispersed in water facilitate integrated reaction and separation. Such integrated methods are important as up to two thirds of total manufacturing costs can be attributed to traditional downstream separation processes [16]. This proof-of-concept work demonstrates that polymer nanoreactors self-assembled by noncovalent interactions are a possible technology for enabling green processing engineering to complement alternative approaches, including membrane reactors [17,18], as well as continuous flow synthesis and nanofiltration [16]. Building on this proof-of-concept study, further future effort is needed to address methods to achieve precipitate with improved purity. Products with higher purity may be possible with a higher temperature and pressure [21] or platinum-based catalysts [34].

Evaluating green chemistry metrics, we examined the E factor. Since we used water as the bulk solvent, the E factor for the reaction itself is relatively low: ~25. The E factor is approximately half of a one-pot synthesis method using a nickel-based catalyst, ethanol as the solvent, and an elevated temperature (105 °C) and pressure (1.4 MPa) [21]. The E factor for the reaction was also comparable to results reported using a bifunctional metal/solid acid catalyst on mesoporous carbon [33].

Depending on the purity of the desired product, the E factor will increase. For example, the extraction of the desired product from the pellet increases the E factor to ~400, although we note that the process was not designed to minimize solvent usage. This result is comparable to previous studies reporting product purification by extraction [33]. Further purification by column chromatography is solvent intensive with E factors of ~10,000–25,000 [35,36]. Clearly, the isolation of the desired reaction product with solvent contributes significantly to the E factor. Therefore, performing multiple reaction steps in “one pot” to avoid the product isolation of intermediate steps would be beneficial for reducing the waste associated with liquid phase chemical processing.

We further analyzed the effect of the cascade reaction steps on the stability of the polymer nanoreactors. Stability was defined as maintaining a hydrodynamic diameter and PDI within 25% of the initial DLS measurement, as long as the PDI remained below a maximum value of 0.400. As can be seen in Table 3, the nanoreactor size and polydispersity was not affected by the first reaction dispersion or pH adjustment. Following the second reaction (imine formation), there were multiple peaks apparent by dynamic light scattering and a significant increase in PDI. This increase in PDI suggests a broad distribution of large aggregates [37]. We attributed this increase in PDI to precipitation of the desired reaction product. To confirm that the nanoreactors were stable following the reaction, we centrifuged the dispersion following the second reaction to separate the precipitated product from the nanoreactor dispersion. We analyzed the resulting supernatant by DLS. After centrifugation to remove the precipitated product, the original size and PDI of the nanoreactor were recovered. Specifically, there was only one peak by DLS that corresponds to a diameter around the initial size of the nanoreactors, and the PDI again falls below 0.400. This result suggests that the nanoreactors are stable throughout both steps of the reaction. To support this analysis, following centrifuging, we analyzed the pellet for gold using ICP-OES analysis. Minimal gold was detected in the pellet, suggesting that the nanoreactors were stable during both steps of the reaction (Table 2). Since the product spontaneously phase separates from the nanoreactors, this is a promising approach to ease reuse of the catalyst.

Finally, we were interested in comparing the performance of the polymer nanoreactors with PEG-coated gold nanoparticles and citrate-stabilized gold nanoparticles in the one-pot reaction. Performing the one-pot reaction with either PEG AuNP and citrate AuNP yielded ~1.5 mg of product which is comparable to the polymer nanoreactors (Table 2). However, both yielded more of the undesired product than the polymer nanoreactors (statistically significant with 90% confidence). Thus, the fraction of product that made up the precipitate was approximately twofold higher for the amphiphilic, self-assembled nanoreactors than the citrate-stabilized gold nanoparticles or PEG-stabilized gold nanoparticles. This result demonstrates that the amphiphilic structure of the self-assembled polymer nanoreactor enhances the performance compared to small molecule or hydrophilic polymer stabilizers. This enhancement is consistent with studies and has been attributed to reactant solubility in the hydrophobic core of the nanoreactor [20].

Interestingly, ICP-OES analysis of the precipitate resulting from the one-pot reactions using PEG AuNP and citrate AuNP reactions indicated that a significant amount of gold precipitated out of the reaction solution (3.6 ± 0.2 and 4.3 ± 0.4 μg, respectively) (Table 2). In contrast, minimal precipitation (<0.9 μg) was observed in the one-pot reaction when using the nanoreactors. These results suggest that the ligand-stabilized (PEG and citrate) gold nanoparticles were less stable than the polymer nanoreactors. Specifically, the amount of gold retained in the dispersion using the self-assembled polymer structures was improved approximately fourfold compared to alternative ligand-stabilized gold nanoparticles. This lack of stability of ligand-stabilized gold nanoparticles in the two-step reaction could be due to removal of the stabilizing ligands by sodium borohydride [24], resulting in colloidal instability.

Notably, while PEG AuNP and citrate AuNP yielded comparable amounts of product as the nanoreactors, there was significant gold in the precipitate as measured by ICP-OES, indicating that these systems are not stable during the cascade reaction and precipitate with the product. This would complicate both isolation of the product as well as reuse of the catalyst. Therefore, the block-copolymer-stabilized nanoreactors are a promising approach for reuse of the catalyst since the product spontaneously phase separates from the nanoreactors without contamination by the catalyst. Taken together, these results indicated that the product spontaneously separated from the reaction mixture, and the nanoreactors were stable in the dispersion. Therefore, the nanoreactor dispersions could be recycled. Micelle-based nanoreactors have been recycled using this approach, which has been demonstrated [38,39]. Future work to understand the recyclability of the nanoreactors, i.e., the performance with multiple reaction cycles, is a key future direction for understanding the functional limitations of the nanoreactors. Further studies to understand the material selection with respect to reactants, products, and polymer nanoreactor components that facilitate the reaction and phase separation would also be valuable.

## 4. Conclusions

In this proof-of-concept study, we demonstrate that amphiphilic polymer nanoreactors dispersed in water can facilitate the two-step imine production with spontaneous phase separation of the product from the reaction mixture. Specifically, we incorporated gold nanoparticle catalysts in amphiphilic polymer nanoreactors using flash nanoprecipitation, a scalable, room temperature process. We use the polymer nanoreactors to perform one-pot condensation of benzaldehyde with 4-nitrophenol performed in water at ambient conditions. The desired 4-benzylideneaminophenol product spontaneously precipitated from the reaction mixture, while the nanoreactors remained stable in dispersion. The turnover frequency was 9 h^−1^, comparable to previous reports. The yield was 65%. Importantly, the self-assembled, amphiphilic nanoreactor structure provided improved performance compared to citrate-stabilized gold nanoparticles and PEGylated gold nanoparticles. The purity of the precipitated product was twofold higher for the nanoreactors compared to the other ligands. The stability was also improved; fourfold more gold was retained in the dispersion. Overall, this work demonstrates that amphiphilic polymer nanoreactors that can be self-assembled using flash nanoprecipitation with modular selection of the nanoreactor components is a promising approach for combined reaction and separation and could be a valuable tool in green process engineering.

## Figures and Tables

**Table 1:** 4-Nitrophenol reduction rate constants of polymer nanoreactors and gold colloids using a 4-nitrophenol concentration of 7.2 mM and a sodium borohydride concentration of 0.1 M (*n* = 3).

Catalyst	k_1_ (L m^−2^ s^−1^)
PS 7500 NR	0.07 ± 0.01
PEG AuNP	0.10 ± 0.01
Citrate AuNP	0.03 ± 0.01

**Table 2:** Analysis of precipitate from cascade reaction (*n* = 3).

	Isolated Product (mg)	Other Solid (mg)	Gold in Precipitate (μg)
PS 7500 NR	1.8 ± 0.1	2.4 ± 1.0	<0.9.
PEG AuNP	1.5 ± 0.5	4.9 ± 1.7	3.6 ± 0.2
Citrate AuNP	1.4 ± 0.3	5.3 ± 1.9	4.3 ± 0.4

**Table 3:** DLS measurements of nanoreactor reaction solution throughout the one-pot reaction. (*n* = 4).

Cascade Step	Peak 1 (nm)	Peak 2 (nm)	PDI
Before Rxn	91 ± 5		0.163 ± 0.018
After 4NP Reduction	98 ± 6		0.133 ± 0.014
After Neutralization	114 ± 25		0.177 ± 0.001
After Imine Formation	461 ± 198	47 ± 8	0.660 ± 0.235
After Centrifuge	104 ± 6		0.139 ± 0.012

## Data Availability

Not applicable.

## Supplementary Materials

The following are available online at https://www.mdpi.com/article/10.3390/polym13121992/s1, Figure S1. Representative image of ^1^H-NMR spectra for the product 4-benzylideneaminophenol.
