# Supplementary material for: Amphiphilic Polymer Nanoreactors for Multiple Step, One-Pot Reactions and Spontaneous Product Separation
Source: Polymers (Basel). 2021 Jun 18;13(12):1992. doi: 10.3390/polym13121992 (PMC8234837; doi:10.3390/polym13121992)
Supplement: Supplementary file 1 [file polymers-13-01992-s001.zip › polymers-1239123-supplementary.pdf]

Supplementary Information

Amphiphilic Polymer Nanoreactors for Multiple Step, One-Pot Reactions and Spontaneous Product Separation

Andrew Harrison [1] and Christina Tang [1,*]

[1] Virginia Commonwealth University, Department of Chemical and Life Sciences Engineering, Richmond, VA 23284-3028, USA
* Correspondence: ctang2@vcu.edu (C.T.)

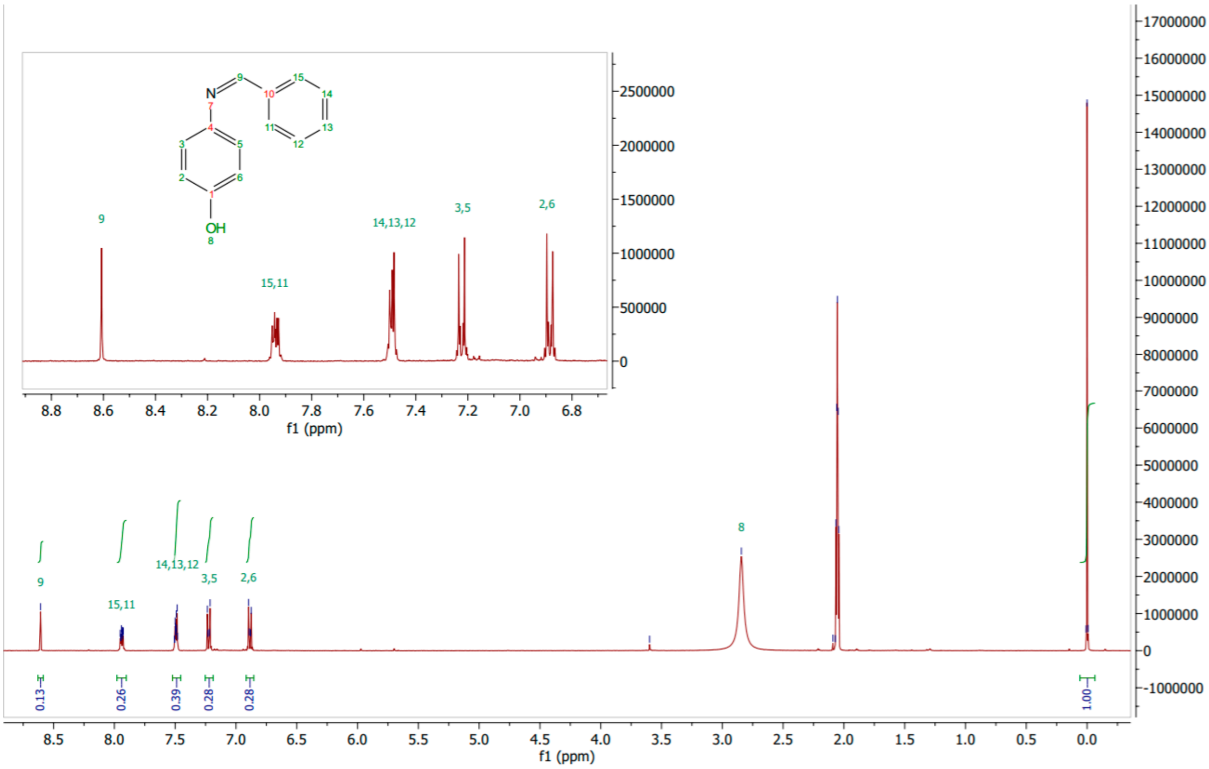

Figure S1: Representative image of ^{1}H-NMR spectra for the product 4-benzylideneaminophenol extracted from the precipitate of the reaction using acetone d-6 and analyzed without further purification. The aromatic region shows two sets of doublets 6.9 and 7.2 ppm, consistent with the aromatic protons on the phenol group. A doublet of doublets is seen further downfield consistent with the aromatic protons in the benzyl group. Lastly, the singlet located at 8 ppm which integrates for 1 proton compared to the doublet peaks at 6.9 ppm is consistent with the benzylidene proton. These results are consistent with the desired product 4-benzylideneaminopheno indicating the it precipitates from the nanoreactor dispersion.
